# Needs assessment for the creation of a community of practice in a community health navigator cohort

**DOI:** 10.1186/s12913-021-06507-z

**Published:** 2021-07-05

**Authors:** Rachel J. Livergant, Natalie C. Ludlow, Kerry A. McBrien

**Affiliations:** Department of Family Medicine, University of Calgary, Cumming School of Medicine, 3330 Hospital Drive NW, Calgary, AB T2N 4N1 Canada

**Keywords:** Community health navigator, Primary care, Community of practice, Quality improvement, Knowledge exchange

## Abstract

**Background:**

Community Health Navigators (CHNs) are members of a patient’s care team that aim to reduce barriers in accessing healthcare. CHNs have been described in various healthcare settings, including chronic disease management. The ENhancing COMmunity health through Patient navigation, Advocacy, and Social Support (ENCOMPASS) program of research employs CHNs, who are trained to improve access to care and community resources for patients with multiple chronic diseases. With complex and demanding roles, it is essential that CHNs communicate with each other to maintain knowledge exchange and best practices. A Community of Practice (CoP) is a model of situated learning that promotes communication, dedication, and collaboration that can facilitate this communication. The objective of this study was to engage with CHNs to determine how a CoP could be implemented to promote consistency in practices and knowledge for CHNs across primary care sites.

**Methods:**

A needs assessment for a CHN CoP was conducted using sequential steps of inquiry. A preliminary focused literature review (FLR) was done to examine the ways in which other healthcare CoPs have been implemented. Results from the FLR guided the creation of an exploratory survey and group interview with key informants to understand best approaches for CoP creation. Political, economic, social, and technological (PEST) and strengths, weaknesses, opportunities, and threats (SWOT) analyses synthesized results in a comprehensive manner for strategic recommendations.

**Results:**

The FLR identified different approaches and components of healthcare CoPs and guided analyses of mitigatable risk factors and leverageable assets for the intervention. The survey and group interview revealed an informal and effective CoP amongst current CHNs, with preferred methods including coffee meetings, group trainings, and seminars. A well-maintained web platform with features such as an encrypted discussion forum, community resource listing, calendar of events, and semi-annual CHN conferences were suggested methods for creating an inter-regional, formal CoP.

**Conclusion:**

The study findings recognise the presence of an informal CoP within the studied CHN cohort. Implementation of a formal CoP should complement current CoP approaches and aid in facilitating expansion to other primary care centres utilizing digital communication methods, such as a comprehensive web platform and online forum.

**Supplementary Information:**

The online version contains supplementary material available at 10.1186/s12913-021-06507-z.

## Introduction

Patient-level barriers and the complexity of the healthcare system can impede adherence to evidence-based clinical care recommendations known to promote better health outcomes and lower resource use, particularly for patients with multiple chronic conditions [1–4]. ENhancing COMmunity health through Patient navigation, Advocacy, and Social Support (ENCOMPASS) is a research program in Alberta, Canada aimed at determining the effectiveness of Community Health Navigators (CHNs) in improving primary care outcomes for patients with chronic diseases. CHNs function in a patient navigator role that can be filled by Community Health Workers (CHWs); they serve as intermediaries between health and social services, and the community [5–7]. CHNs are overseen in their activities by a licensed social worker, but not regulated by a specific college or administrative body. Although most CHNs possess Bachelors or equivalent degrees, CHNs do not require any previous healthcare-specific training, and they are chosen based on their leadership and communication skills, professionalism, community engagement, and commitment to service [5, 8].

ENCOMPASS is currently partnered with one Primary Care Network (PCN) in Calgary, Alberta, Canada, and is expanding implementation to three additional Albertan PCNs. In Alberta, PCNs bring health care professionals together to provide comprehensive team-based primary care to patients [9]. Ensuring the commitment, proficiency, and dedication of CHNs in their roles in primary care is essential to the success of the ENCOMPASS program. Creating a Community of Practice (CoP) for CHNs is one way to promote best practices and knowledge exchange within the group. As the ENCOMPASS program expands, a CoP can promote consistency in practices and protocols across the program, both among CHNs within a given PCN, and between CHNs from different PCNs across the province.

CoPs are originally based on the theory of situated learning described by Étienne Wenger – a system of colleague interaction –- but the term CoP has evolved into a broader term describing a group of people who share a common purpose and come together to interact, learn, and form a sense of identity [10]. The operation of a CoP is a dynamic process – it promotes and demands continual learning of all members and is a collective responsibility for managing needed knowledge and ensuring the availability of essential resources [11]. Three characteristics define a CoP: a shared domain of interest and commitment to this domain by participants; the creation of a community, wherein the members build relationships that facilitate reciprocal learning; and the development of the practice itself [10]. CoPs can be either informal or formal. Informal CoPs are established naturally, without any deliberate introduction of approaches into a given group [10, 12]. Conversely, a formal CoP involves deliberate implementation of specific approaches with the intent to form an organized CoP [10, 12]. In healthcare settings, both formal and informal CoPs exist that utilize several different approaches to drive knowledge management and improve performance [13]. These approaches include workshops, seminars, active member meetings, emails, bulletins, teleconferences, and web platforms [13]. Web platforms are becoming more prevalent in healthcare-related CoPs to promote relationship formation and reciprocal learning [14–17].

To date, there is a lack of literature describing CoPs in a patient navigator cohort. Additionally, there is no recognised protocol for establishing a CoP in this cohort or in creating a larger CoP to encompass an expanded group. The primary objective of this study was to determine the best approach to implementing a CoP within the CHN cohort to help support knowledge exchange and best practices. A secondary objective was to establish a framework for creating a larger CoP among an expanded CHN group spanning different PCNs, capable of maintaining consistent and evidence-based best practices.

## Methods

### Ethics approval and consent

As this project falls under quality assurance/project evaluation, the Conjoint Health Research Ethics Board (CHREB) at the Cumming School of Medicine, University of Calgary waived the requirement for research ethics review under the TriCouncil Policy Statement 2014 (Chapter 2, Article 2.5). The CHREB approved the verbal consent by participants, as participants were involved only in surveys and interviews, not subject to direct interventions, and no identifying information was recorded. Verbal consent from all participants was collected and documented by researchers in a secure database prior to beginning the study. All methods were carried out in accordance with institutional guidelines and regulations.

### Study design

We used sequential steps of inquiry in this needs assessment for CHNs in order to explore and determine how best to introduce a formal CHN CoP. The first step was a focused literature review (FLR) to establish current practices in healthcare CoPs and guide survey development. The survey was used to extract preliminary data from the CHNs on their current and preferred future approaches of communication and knowledge exchange. The final step of data collection was a semi-structured group interview with CHNs that drew on results from the FLR and survey to guide question formation. Results from activities were then compiled into analysis matrices to systematically present recommendations based on the needs assessment, considering both external and internal risks and assets of the intervention.

### Focused literature review

The FLR was conducted to identify CoP approaches used in other healthcare settings. This review focused on quantitative and qualitative studies as well as literature reviews published between January 1, 2009 and January 1, 2019. We searched five electronic databases (MEDLINE, CINAHL, PsycINFO, EMBASE, and SocINDEX) for articles published in English that met the inclusion and exclusion criteria, as described below. The year 2009 was set as the starting point for this review as a comprehensive systematic review of healthcare CoPs included papers up to 2009 [13].

We used a broad set of search terms to represent CoP: *community/communities of practice, community/communities of learning, community/communities of knowledge, community/communities of interest* or *situated learning.* These search terms were combined with terms related to healthcare and medicine (*allied health, medicine/medic/ medical, health/ healthcare/health care*) to narrow the scope of papers for review. Only papers published in peer-reviewed journals were considered. Papers were excluded if they reported studies on CoPs in non-healthcare settings or if the CoP was not about care delivery, such as those focused on medical education, community-based learning, or the pharmaceutical industry. Papers that did not detail the components of the CoP were also excluded.

From the included papers, we extracted data relevant to the study and captured information on locations of CoPs, participating members and groups, the domain of the CoP, and approaches to knowledge exchange and communication.

### Survey

We created a 17-point online survey that was distributed to the CHNs through the survey generator, SurveyMonkey™ (Additional File 1). The CHN manager and six CHNs already involved in the program were invited to participate. Three additional CHNs had not completed training nor interacted previously with the CHN group and therefore were not invited to complete the survey. The survey aimed to broadly define which CoP approaches the CHNs currently use and inquired about future preferences and a CHN web platform:

1. **Current communication and learning approaches.** CHNs were asked to choose which communication approaches were currently used in their group and how helpful and useful the approaches are for the group.
2. **Communication preferences.** Participants were given the opportunity to identify other communication approaches they would like to incorporate into their cohort.
3. **Current knowledge exchange opportunities and preferences.** CHNs were asked to describe desirable topics, frequency, and methods of knowledge exchange, both current and in the future.
4. **Expansion of CHN communication and knowledge exchange.** Participants were asked to assess their comfort level and preferences for expanded communication groups and knowledge exchange activities as more CHNs in other PCNs become incorporated into the CoP.
5. **CHN**-**specific web platform.** We asked CHNs if they had interest in a CHN-specific web-platform, and to brainstorm components of a web-platform that would be beneficial to the group and their roles as CHNs.

### Group interview

Considering the survey responses, we created semi-structured questions for the group interview (Additional File 2). The CHN manager and all nine CHNs were invited to participate in the group interview, as all CHNs had begun interacting together by this time. Only one CHN was not present at the interview. There were three parts to the group interview, which aimed to expand on and clarify answers from the survey. Part one focused on current communication approaches among the cohort, including associated benefits and challenges. This portion also included a discussion about future communication avenues within a given PCN and across multiple PCNs. Part two of the group interview focused on knowledge exchange strategies, and, similar to part one, addressed preferred and less preferred current and future methods for a CoP. The final part of the interview assessed the potential for a CHN web platform. CHNs were provided with sample CoP web platforms and asked to inspect each before the group interview. The interview addressed accessibility, usability, likes, and dislikes of the example web platforms, and desired components for a CHN web platform.

### Analysis

Quantitative data from close-ended survey questions were analyzed descriptively with frequency metrics. We used content analysis to analyze qualitative data from both the survey and the group interview. Information was coded into sections based on topic (communication, knowledge exchange, web platform), and separated into sub-nodes of “current approaches” and “future approaches” for each topic. Sub-nodes were further segmented based on themes of preferences, challenges, and opportunities within each sub-node. A matrix was produced with themes derived from each code (Additional File 3).

Compiled results from the FLR, survey, and group interview were used to guide the completion of Strengths, Weaknesses, Opportunities, and Threats (SWOT) and Political, Economic, Social, and Technological (PEST) analyses. This method of SWOT analysis has been validated as a methodological approach in healthcare, while PEST analyses have been recommended as opportunities to improve strategic analyses in healthcare practices [18].

## Results

### Focused literature review

**Fig. 1:**
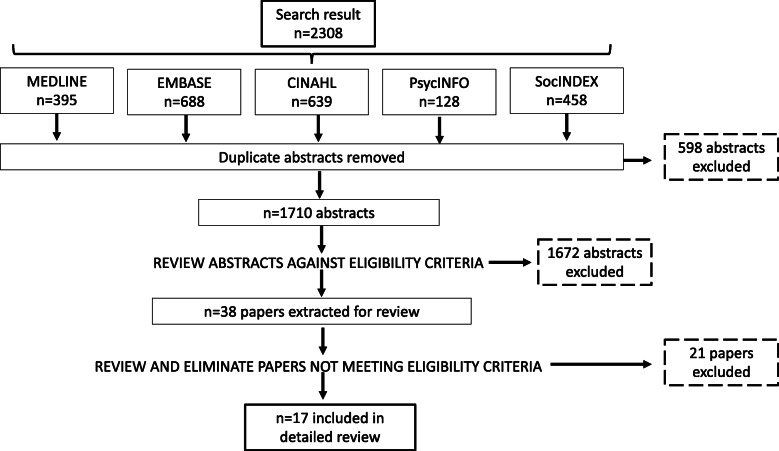
Focused literature review flow diagram

Our search yielded 2308 abstracts from five databases, 598 of which were duplicates, leaving 1710 abstracts for review (Fig. 1). After title and abstract review, 38 papers underwent full text review, and 17 met inclusion criteria (Fig. 1). Two studies originated from Australia [19, 20], eight from Canada [21–28], four from Europe (Spain, United Kingdom, Norway, Sweden, Netherlands) [29–32], and three from the United States of America (USA) [33–35] (Table 1). Practice areas of focus included audiology, geriatric care, occupational therapy, oncology, oral medicine, physiotherapy, primary care, social work for palliative care, and stroke care (Table 1). Ten of the 17 papers described the creation of multidisciplinary/interdisciplinary CoPs, while the other seven papers examined CoPs within one distinct healthcare professional group (Table 1). No papers described CoPs dedicated to patient navigators or members of similar occupations.

**Table 1:** Summary of healthcare CoP studies included in focused literature review

***Paper***	***Title***	***Members***	***Health care domain***	***Location***	***Methods***
**Behl et al 2015 **[35]	The Value of a Learning Community to Support Telepractice for Infants and Toddlers with Hearing Loss	Administrators and clinicians (audiologists)	Audiology	USA (UT)	Initial in-person meeting; monthly 90-min teleconferences (Adobe or traditional), Moodle, Google Docs
**Fingrut et al 2018** [23]	Building an oncology community of practice to improve cancer care	Family physicians, specialists, nurses, allied health professionals	Oncology	Canada (ON)	Meetings, seminars, roundtable discussion
**Fung-Kee-Fung et al 2014** [21]	Exploring a “community of practice” methodology as a regional platform for large-scale collaboration in cancer surgery—the Ottawa approach	Surgeons, oncologists, nurses, social workers, other physicians, public health leaders	Oncology	Canada (ON)	Workshops, regular in-person meetings, video conferences, seminars and presentations, steering committee
**David et al 2012** [27]	Clinicians’ expectations of Web 2.0 as a mechanism for knowledge transfer of stroke best practices	Occupational therapists, nurses, physiotherapists, specialists, speech pathologists, social workers	Stroke Care	Canada (QC)	Web platform (blogs, wikis, podcasts, discussion forums, virtual library)
**Wieringa et al 2018** [32]	How knowledge is constructed and exchanged in virtual communities of physicians: qualitative study of mindlines online	General practitioners	Primary Care	UK, Norway, Netherlands	Web platforms (Facebook, discussion forums)
**Tintorer et al 2015** [29]	Understanding the discriminant factors that influence the adoption and use of clinical communities of practice: the ECOPIH case	Physicians, nurses	Primary and Specialist Care	Spain	Web platform (online discussion forums, social media), cellphone communication
**Jeffs et al 2016** [24]	Contextualizing learning to improve care using collaborative communities of practices.	Nurses, occupational therapists, physiotherapists	Primary and Specialist Care	Canada (ON)	Learning modules, monthly meetings, symposiums
**Hurtubise et al 2016** [28]	Virtual knowledge brokering: describing the roles and strategies used by knowledge brokers in a pediatric physiotherapy virtual community of practice	Physiotherapists	Pediatric Physiotherapy	Canada (QC)	Private access web-platform (online forum, shared resources and clinical tools, links to documents and web links), 2 1-day workshops
**Boucher and MacIntyre 2015** [22]	Survey of a Pelvic Health Physiotherapy Community of Practice: A Pilot Study to Gain Member Input to Help Sustain and Advance the Group	Physiotherapists	Physiotherapy (Pelvic Health)	Canada (AB)	Regular in-person meetings, interest in web platform and social media
**Cassidy 2011** [34]	Online Communities of Practice to Support Collaborative Mental Health Practice in Rural Areas	Nurses	Primary Care (Rural)	USA (TN)	Web platform
**Francis-Coad et al 2018** [19]	Evaluating the impact of a falls prevention community of practice in a residential aged care setting: a realist approach	Allied health professionals, care deputy care managers	Geriatric Care	Australia	In-person meetings, emails, electronic discussion boards, posters/checklists
**Friberger and Falkman 2013** [30]	Collaboration processes, outcomes, challenges and enablers of distributed clinical communities of practice	Dentists, pathologists, oral surgeons	Oral Medicine (Dentistry and Oral Surgery)	Sweden	Monthly teleconferences, case submissions via web platform
**Hoffmann et al 2011** [20]	Evaluating an online occupational therapy community of practice and its role in supporting occupational therapy practice	Occupational therapists	Occupational Therapy	Australia	Web platform (online forums, special interest discussions), occasional in-person meetings
**Kislov et al 2012** [31]	Managing boundaries in primary care service improvement: A developmental approach to communities of practice	General practitioners, nurses, practice manager, specialists	Primary Care (Chronic Kidney Disease)	UK	In-person meetings, informal exchanges, quarterly seminars, teleconferences, web platform
**Kitto et al 2018** [25]	What’s in a name? Tensions between formal and informal communities of practice among regional subspecialty cancer surgeons	Surgical oncologists	Oncology	Canada (ON)	In-person meetings, teleconferences, email, websites, conferences, seminars, informal meetings
**Kothari et al 2015** [26]	Communities of practice for supporting health systems change: a missed opportunity	Nurses, dentists	Oral Medicine (Geriatric)	Canada (ON)	Webinars, in-person meetings, newsletters, online forums, online workshops
**Murty et al 2012 **[33]	Using a LISTSERV to Develop a Community of Practice in End-of-Life, Hospice, and Palliative Care Social Work	Social workers	Social Work (Palliative Care)	USA	Electronic discussion group (LISTSERV), email

With respect to approaches used to form and maintain a CoP, 10 papers described using in-person meetings, workshops, and/or seminars; 13 described the use of a web platform or online forums; and eight papers described the use of teleconferences and/or emails for communication. Use of multiple approaches for communication and knowledge exchange between participants in CoPs were described in 13 papers. Of the four studies that only mention a single CoP approach, CoPs used web platforms that incorporated different components such as blog posts, discussion forums, and resource sharing. Virtual CoPs that allowed for reciprocal and tactic knowledge exchange were the most popular for CoP members [20, 27, 28, 31, 34]. However, teleconferences and in-person, large member meetings and conferences were described as beneficial to CoP members when establishing new guidelines and evolving practices [19, 21, 22, 24, 30, 35].

### Survey and group interview

Results from the FLR targeted three key foci for the CHN CoP survey and group interview: communication preferences, knowledge exchange, and web platform design.

#### Communication

**Fig. 2:**
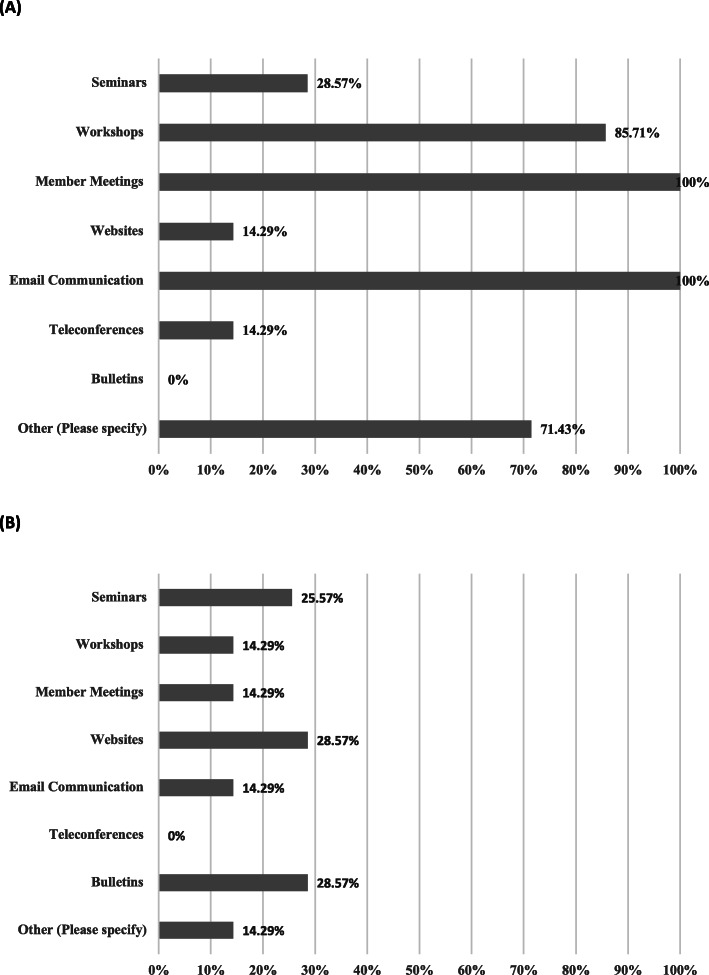
Results from initial community health navigator survey. Survey indicates results for **a** current communication methods and **b** additional methods desired for communication and knowledge exchange within a future community health navigator community of practice

Both the survey and group interview elicited current and preferred future communication approaches in the CHN cohort. From the survey, communication approaches reported as currently in use were seminars (2/7; 28.57%); workshops (6/7; 85.71%); member meetings (7/7; 100%); website (1/7; 14.29%); email communication (7/7; 100%); teleconferences (1/7; 14.29%); bulletins (0/7; 0%); and other (5/7; 71.43%) including group messaging/team text groups (4/7; 57.14%) and coffee/informal meetings (2/7; 28.57%) (Fig. 2a). Respondents indicated that future communication approaches for a CoP should include seminars (2/7; 28.57%); workshops (1/7; 14.29%%); member meetings (1/7; 14.29%); website 2/7; 28.57%); email communication (1/7; 14.29%); teleconferences (0/7; 0%); bulletins (2/7; 28.57%); and other (1/7; 14.29%), in which the respondent indicated no desire for new approaches (Fig. 2b). Overall, the following rankings were given to different modalities of communicating, listed in order of most preferred to least: in-person; email; web chatting; and phone.

The group interview further categorized current communication approaches into informal and formal categories. Formal communication approaches include those that are mandatory or officially organized for the CHNs for work purposes. Informal approaches involve communication arranged personally or spontaneously by or for the CHNs. Informal approaches like coffee dates and group text messages, both between small groups and the larger CHN group, were reported as useful for information exchange and maintaining relationships. With regards to formal communication approaches, CHNs appreciated their planned member meetings, which tend to be casual, allowing personal connections to form and greater comfort in discussing patient care. Initial training sessions as well as organic discussion within member meetings keep CHNs motivated and engaged in the program. Formal emails between the CHNs and ENCOMPASS researchers were described as challenging and confusing due to long and sometimes convoluted threads. For future communication, the CHNs liked the idea of frequent networking with other healthcare providers and organizations to build interdisciplinary relationships. Overall, communication was deemed essential to the CHNs, due to isolation felt within their role. The CHNs therefore emphasized a continued need for frequent communication with other CHNs, whether in person or over text messaging, within a CoP.

### Knowledge exchange

In both the survey and group interview, CHNs reported that opportunities to participate in knowledge exchange primarily involve member meetings and group chats. Onboarding training was valuable to the CHNs, as well as continuing opportunities for training and education that occur through online modules, research articles, and news emailed by their manager. Although these resources are appreciated, they often are not easy to access as they are stored in email threads. Linking resources in a permanent space and incorporating other contributors to resources were suggested as potential improvements.

Current knowledge exchange with each other was reported as satisfactory but the CHNs acknowledged that future expansion may require additional approaches. A mini-conference quarterly or semi-annually between all ENCOMPASS CHNs with presentations and workshops from both CHNs and other healthcare professionals and organizations was suggested as an opportunity to foster in-person relationships that allow for better engagement and knowledge exchange between CHNs from other PCNs.

### Web platform

The final portion of the survey and group interview examined preferences and perceived challenges of an ENCOMPASS PCN-wide CHN web platform. In the survey, 6/7 (85.61%) respondents expressed interest in a web platform. When asked why a web platform would be beneficial, CHNs indicated the value in a “one-stop shop” for things like resources, forums, and trainings. The respondent who answered ‘no’ did not provide a reason for their response. Components of a CoP web platform considered potentially valuable included a list with links to useful resources in the community that are commonly needed for the patient population; a discussion forum; problem scenarios with clients/physicians and troubleshooting for these issues; and a list of links to health resources.

In the group interview, participants indicated an interest in a web platform, however, only if it is maintained weekly with relevant information. After viewing other CoP web platforms, the CHNs had several recommendations for their own web platform. Components such as a private-access online forum, community resources, calendar of events for patients to attend in relevant regions, links to online trainings, applicable news and research, and an overview of common diseases and medications taken by their patient population were requested additions. CHNs also expressed a desire to keep their forum private from other healthcare professionals to avoid confounding opinions. However, the CHNs recommended having a public web page explain their function to help legitimize their role to other healthcare providers and patients. Most of the CHNs found the sample web platforms to have unnecessary components and barriers to use. A web platform that is uncluttered and contains only information that is applicable to CHNs and their roles would increase its accessibility. As well, CHNs preferred limited usability barriers, such as multi-step logins to portals, as this is seen as a significant obstacle to web platform use. Finally, CHNs had significant concerns regarding patient confidentiality, which they fear could be breached in an online discussion forum.

### PEST and SWOT analyses

**Fig. 3:**
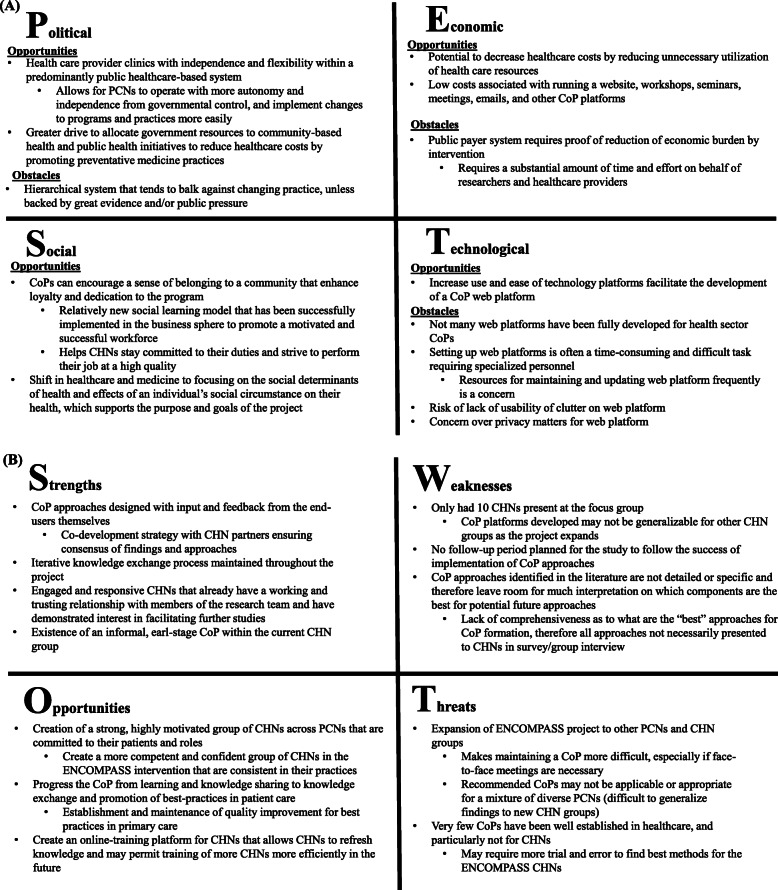
Strategic analyses of a community health navigator community of practice. **a** Political, economic, social, and technological (PEST) and **b** strengths, weaknesses, opportunities, and threats (SWOT) analyses based on findings from the focused literature, survey, and group interview. CHN: Community Health Navigator; PCN: Primary Care Network

PEST and SWOT analyses were performed using information gathered from the literature review, background scan, and survey and group interviews (Fig. 3). These analyses highlighted factors to mitigate and leverage for the success of an inter-organizational CHN CoP and helped shape the recommendations stemming from this study.

There are several beneficial factors inherent to the CHN CoP study and its design that can be leveraged to promote the success of the program. The CHN group involved in this study are a very engaged and motivated group with a good pre-existing working relationship with the research team. Additionally, the presence of an informal CoP within the MPCN CHN, coupled with the co-creation strategy of recommendations with CHN end-users, makes adoption of new CoP approaches more likely and may facilitate uptake of recommendations into other PCNs with CHNs working in similar roles.

External factors can likewise be leveraged to aid in the success of the CHN CoP. Firstly, Albertan PCNs have more independence and flexibility to decide which programs they wish to implement, compared to other public models of primary care in the province [9]. Additionally, inexpensive and cost-effective interventions, such as creation of a CoP, are more likely to be embraced by the healthcare system [36]. Secondly, using technology to build and maintain the CoP allows for easy portability of the CoP, especially as the ENCOMPASS program expands to other PCNs. Finally, the structure of CoPs create beneficial social results by promoting a sense of community and motivation in members, leading to greater job satisfaction and incentive to continue interacting within the CoP.

Several risks were identified internal to the CHN CoP program, that should be considered and mitigated during CoP implementation. Only 10 CHNs from one PCN gave input on preferences and dislikes regarding CoP approaches. As the ENCOMPASS program expands to other PCNs, this may result in lack of generalizability and difficulty in adoption of the CoP by other CHN groups. Additionally, the literature surrounding strategies for CoP implementation and details on CoP methods is sparse, limiting the breadth of approaches presented to CHNs during the course of this study. Furthermore, this is the first CoP to our knowledge being implemented in a CHN cohort, so there remains uncertainty surrounding its applicability and effectiveness in improving CHN care capacity, and consequently patient outcomes.

Other broad, systems’ level risks that may impede success of the CoP include: the hierarchical medical system that is slow to adopt changes [37]; burden on researchers to prove economic feasibility of the program for widespread adoption [36]; and accessibility, barrier, and usability issues inherent to establishment of web platforms and other technology-based approaches.

## Discussion

We performed a comprehensive needs assessment for the implementation of a CoP for a CHN cohort. Our FLR, survey, and group interview with CHN stakeholders led to several recommendations on best approaches for communication, knowledge exchange, and web platform creation to include in a formal patient navigator CoP (Table 2). Furthermore, findings from this study include a suggested implementation strategy for the CoP to improve likeliness of success and adoption.

**Table 2:**
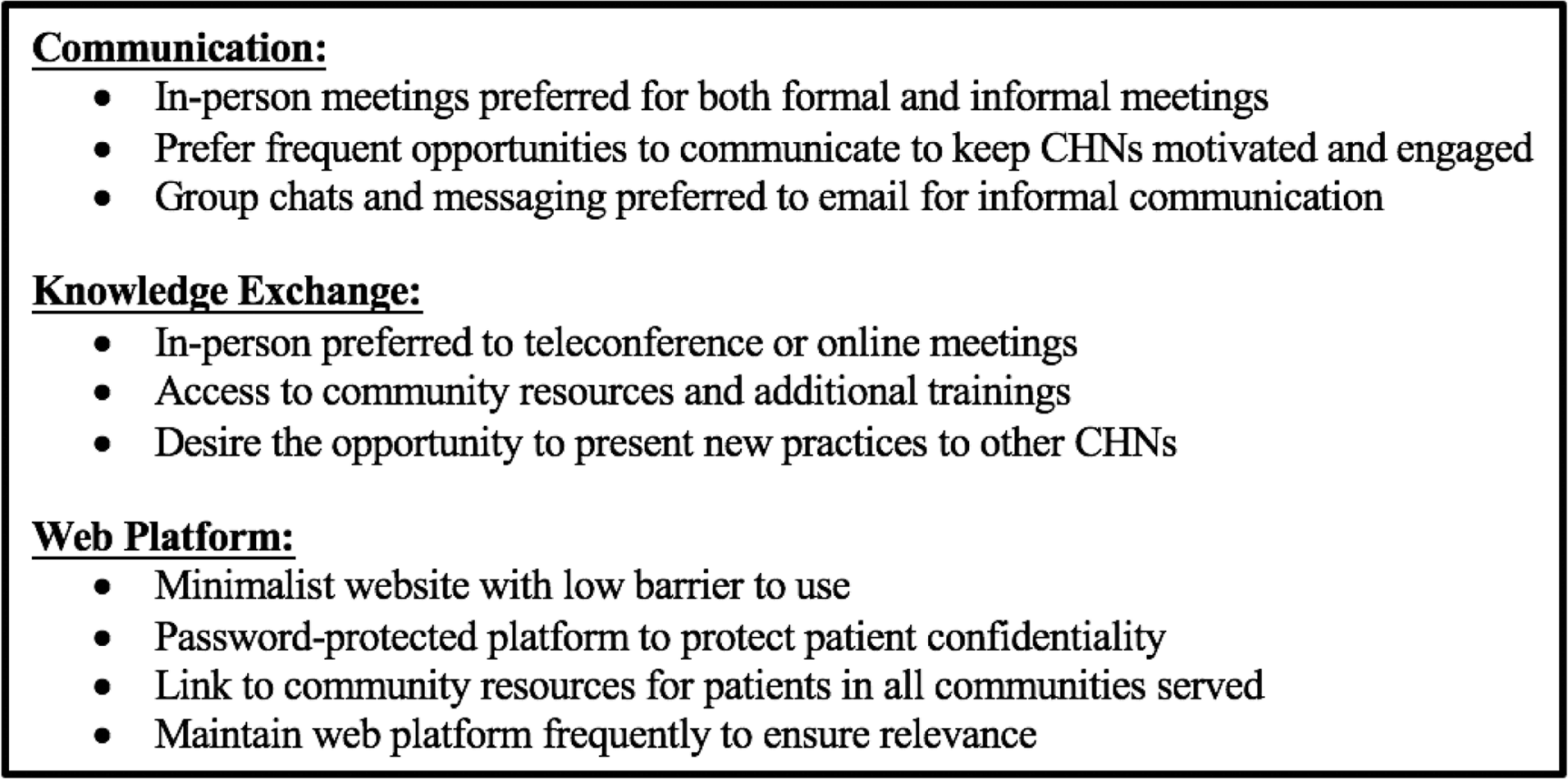
Strategic recommendations for implementing a community of practice in a community health navigator cohort. CHN: Community Health Navigator

The CHN cohort currently employs several informal CoP approaches such as in-person meetings, group chats, workshops, and trainings. According to CHNs, these foster a sense of comradery and allow for a close social network between members that facilitates sharing new knowledge, concerns, and challenges. When looking to create a more formal CoP within this CHN group, these current informal CoP approaches should not be replaced. Replacing informal CoPs with formal ones can lead to tensions and disengagement of members from the CoP [10]. Therefore, additional CoP approaches, such as a web platform and conferences, should complement and not replace existing approaches.

For communicating and knowledge exchange opportunities, the CHNs preferred in-person meeting and frequent touch points to teleconferencing or online webinars. Although it has been proposed that in-person meetings facilitate greater engagement by participants, these are not always feasible given geographic, environmental, or other unpredictable constraints, as evidenced by the coronavirus disease 2019 (COVID-19) pandemic [38]. Therefore, although all efforts should be made to provide in-person meetings and conferences, contingencies should be in place to host virtual events. To increase satisfaction with virtual communication and knowledge exchange, synchronous conferencing with virtual collaboration should be encouraged for best results [39]. To prevent common pitfalls associated with virtual platforms and avoid technological obstacles identified in the PEST analysis, technology should be properly tested and have a robust and scaled support program to ensure smooth operation [40].

A web platform can help bolster communication and knowledge exchange approaches for CHNs and was indicated as being a valuable addition by the CHN cohort. Several web platforms currently exist to support healthcare CoPs [41–45], but these platforms presented various limitations and barriers to use that were objectionable to the CHNs. Robust usability and accessibility testing of a future web platform should therefore be conducted with CHNs to ensure satisfaction and increase use. In particular, CHNs were concerned about communication and exchanging potentially sensitive patient information on a non-secure platform. Balancing privacy and patients’ needs in the age of online engagement has been a challenge for many healthcare professionals, however, following guidelines such as removing patient identifiers and ensuring a secure, encrypted discussion forum are methods for protecting patient privacy and CHNs from liability [46].

### Limitations

These findings should be interpreted with respect to limitations of this study. The scope of this study is limited, due in part to the lack of available published literature on the topic and the low number of participants included in the study. Given the considerable lack of published literature on CoPs being implemented in a CHN or CHW cohort, approaches for CoP formation were recommended based on CoPs for other healthcare providers. Additionally, due to the limited number of CHNs engaged with the ENCOMPASS study, we were only able to sample opinions from six participants in the survey, and nine participants in the group interview. Consequently, the generalizability of the results of this study to wider populations is unknown and should be the focus of follow-up studies.

## Conclusion

This needs assessment is first known study to provide a detailed evaluation and implementation strategy for a CHN CoP. The current approaches to communication and knowledge exchange amongst CHNs denote the presence of an informal CoP. These approaches may be effective in other CHN groups from other PCNs and regions and should be proposed to managers responsible for the new cohorts. As the ENCOMPASS research program expands, it becomes difficult to maintain consistency of these informal approaches for broader CHN communication and knowledge exchange. A formal CoP should be implemented for inter-regional communication and collaboration. Overall, when establishing a CHN CoP, it is important to consider current approaches and partner with the cohort to make improvements and implementing additional approaches, fostering the creation of a strong, comprehensive CoP. This methodology can be extrapolated to other health worker groups. A similar needs assessment can help implement CoPs in these cohorts for improved communication and knowledge exchange, and consequently, has the potential for supporting superior healthcare programs.

## Supplementary Information

**Additional file 1.** Survey questions for the community health navigator cohort community of practice needs assessment. This file contains the survey sent out to community health navigators at the beginning of the study to ascertain current approaches to communication and knowledge exchange. Additionally, this survey probed community health navigators to define preferences for future approaches to communication, knowledge exchange, and a web platform for an upcoming community of practice.

**Additional file 2.** Group interview questions for the community health navigator cohort community of practice needs assessment. This file contains the format and questions from the group interview with community health navigators used to elaborate on current and preferred future approaches to communication and knowledge exchange. The main portion of the group interview focused on preferences and objections for components of future a web platform for the future community of practice.

**Additional file 3.** Inductive and deductive qualitative and narrative data synthesis from the group interview. This table provides a summary and examples of the thematic analysis conducted to.

## Data Availability

The datasets used and/or analysed during the current study available from the corresponding author on reasonable request.

## Abbreviations

CHN: Community Health Navigator
CoP: Community of Practice
ENCOMPASS: ENhancing COMmunity health through Patient navigation, Advocacy, and Social Support
PCN: Primary Care Network
