# Supplementary material for: Needs assessment for the creation of a community of practice in a community health navigator cohort
Source: BMC Health Serv Res. 2021 Jul 5;21:657. doi: 10.1186/s12913-021-06507-z (PMC8256652; doi:10.1186/s12913-021-06507-z)
Supplement: Supplementary file 1 — Additional file 1. Survey questions for the community health navigator cohort community of practice needs assessment. This file contains the survey sent out to community health navigators at the beginning of the study to ascertain current approaches to communication and knowledge exchange. Additionally, this survey probed community health navigators to define preferences for future approaches to communication, knowledge exchange, and a web platform for an upcoming community of practice. [file 12913_2021_6507_MOESM1_ESM.pdf]

Additional File 1. Survey questions for the community health navigator cohort community of practice.

Q1. Which of the following approaches are currently used by your CHN group to facilitate communication and learning?

- **a.** Seminar:
- **b.** Workshop:
- **c.** Member Meetings:
- **d.** Websites:
- **e.** Email Communication:
- **f.** Teleconferences:
- **g.** Bulletins (Monthly/Weekly Updates)
- **h.** Other (please specify)

Q2. Please explain what is helpful (or not helpful) about these approaches.

Q3. Which of the following methods would you like to see incorporated into your CHN group to facilitate communication and learning? Please select all that apply.

Q4. What has been the most useful method of communication and knowledge sharing for your CHN group, in your opinion? Please state why.

Q5. How do you prefer to communicate and learn?

Q6: How often would you like to attend in-person meetings, workshops and/or seminars?

Q7: When you or someone else in the group learns new ways to perform a specific task more easily or better than previously, how is this information shared with the rest of the group?

Q8. Would you be interested in on-line training resources?

Q9. Please explain your answer to question 8 here.

Q10. What training resources would you like to be able to access online?

Q11. What topics would you like covered in in-person seminars and/or workshops?

Q12. Would you feel comfortable with sharing your experiences and knowledge gained in your job with other CHNs from other PCNs or organizations?

Q13. Please explain your answer to question 12 here.

Q16. Are you interested in a CHN-specific website that provides a space for CHNs to communicate and share resources with one another?

Q15. What elements and components would like to see in a CHN- specific website?

Q16. Please explain your answer to question 15 here.

Q17. Do you have any other comments for us at this time related to the creation of a Community of Practice for CHNs in Alberta?
