# Supplementary material for: Needs assessment for the creation of a community of practice in a community health navigator cohort
Source: BMC Health Serv Res. 2021 Jul 5;21:657. doi: 10.1186/s12913-021-06507-z (PMC8256652; doi:10.1186/s12913-021-06507-z)
Supplement: Supplementary file 2 — Additional file 2. Group interview questions for the community health navigator cohort community of practice needs assessment. This file contains the format and questions from the group interview with community health navigators used to elaborate on current and preferred future approaches to communication and knowledge exchange. The main portion of the group interview focused on preferences and objections for components of future a web platform for the future community of practice. [file 12913_2021_6507_MOESM2_ESM.pdf]

Additional File 2. Group interview questions for the community health navigator cohort community of practice needs assessment.

Part 1: Communication Preferences

Preamble: In this first section, I want to capture your perspective on communication preferences in your role as a CHN. I want to better understand how you all communicate now, and how you think these communication techniques could be expanded as the ENCOMPASS CHN group expands further.

1. How do you, as a group, normally communicate with one another? do you primarily communicate in person, electronically, by phone? Do you use certain forms of communication preferentially depending on what the communication is about?

2. How would you expand communication to a larger group of CHNs not just in Mosaic, especially if frequent in-person meetings aren't possible? How would you share information about resources with another PCN group? How would you find out about how other groups are approaching certain issues?

3. What other forms of communication would you like to have for your group? Are there challenges with how you currently communicate that could be overcome with different modes of communication?

4. Do you use social media platforms to communicate? If yes, which ones and why? If no, why not and do you think any would be helpful?

5. Do you have any other comments or concerns on communication approaches in your CHN group at this time?

Part 2: Learning and Educating Preferences

Preamble: Now I would like to move onto learning and education. Here I am interested in hearing your perspective on current learning resources and how a CoP could support those. I know you recently participated in a focus group about training, so I don't want you to get into the specifics of the training program, but rather how and from where you learn outside of the more formal training.

1. What current opportunities do you have to learn new ways to improve patient management? How might you find out about what other similar programs do for their

patients? How do you find out about new programs/resources available through Mosaic, AHS or the broader community?

2. Community of Practices require both teaching and learning opportunities for all members. How would you like new information on patient care to be presented to you? How would you feel most comfortable presenting new best-practices that you learn?

3. Workshops and seminars have been identified by some of you as potentially valuable learning and knowledge transfer methods for your group. Would you like to run these workshops or would you like experts from other fields to present? What are your thoughts on an ENCOMPASS CHN conference or annual meeting for all CHNs and individuals involved in the ENCOMPASS program in the future?

4. What other resources would you like to support you in your role as a community health navigator?

5. Do you have any other comments or concerns on learning and educating techniques within the CHN-group at this time?

Part 3: Web Platform Development

Preamble: For the last part of this focus group, I am interested in understanding your preferences related to a web-based Community of Practice. I sent out some examples of web-platforms being used by other Community of Practices and want to gauge your opinions on these platforms and how you might like a website for your group to look and what components you'd like to see on one.

1. What do you think about having a central website where you could access information, communicate with each other and other groups, and view opportunities for shared learning etc.?

2. What aspects of the web-platforms that I sent out did you like the best? What aspects did you not like? How easy was it to navigate the web-platforms? What improvements would you like to the usability of a web-platform for yourself?

3. Are there any other aspects of a potential web-platform for your group that you would like included that were not present in the example web-platforms?

4. What type of external resources and links would you like to have available on a CHN platform?

5. Would you like separate sections on the web-platform that are specific for each PCN CHN group? Or would you prefer just one web-platform for all CHNs involved in the ENCOMPASS program?

6. How do you feel about including other healthcare professionals on the ENCOMPASS CHN web-platform?

7. Do you foresee any drawbacks or challenges with a web-platform for the ENCOMPASS CHN-group?

8. Do you have any other comments on the establishment of a web-platform for the ENCOMPASS CHNs at this point?
